# Initiating-clone analysis in patients with acute myeloid leukemia secondary to essential thrombocythemia

**DOI:** 10.1038/s41598-024-66461-8

**Published:** 2024-07-10

**Authors:** Yoko Ushijima, Seara Naruse, Yuichi Ishikawa, Naomi Kawashima, Masashi Sanada, Marie Nakashima, Jeong Hui Kim, Seitaro Terakura, Rika Kihara, Koichi Watamoto, Takahiro Nishiyama, Kunio Kitamura, Tadashi Matsushita, Hitoshi Kiyoi

**Affiliations:** 1 Department of Hematology and Oncology, Nagoya University Graduate School of Medicine, Tsurumai-Cho 65, Showa-Ku, Nagoya, 466-8550 Japan; 2 Clinical Research Center, National Hospital Organization Nagoya Medical Center, Nagoya, Japan; 3 Department of Hematology and Oncology, Konan Kosei Hospital, Konan, Japan; 4 Department of Hematology, Komaki City Hospital, Komaki, Japan; 5 Division of Hematology, Ichinomiya Municipal Hospital, Ichinomiya, Japan; 6 Department of Transfusion Medicine, Nagoya University Hospital, Nagoya, Japan

**Keywords:** Acute myeloid leukaemia, Myeloproliferative disease

## Abstract

Most of essential thrombocythemia (ET) patients have the clone harboring a mutation in one of the *JAK2*, *CALR*, or *MPL* gene, and these clones generally acquire additional mutations at transformation to acute myeloid leukemia (AML). However, the proliferation of triple-negative clones has sometimes been observed at AML transformation. To clarify the clonal evolution of ET to AML, we analyzed paired samples at ET and AML transformation in eight patients. We identified that *JAK2*-unmutated AML clones proliferated at AML transformation in three patients in whom the *JAK2*-mutated clone was dominant at ET. In two patients, *TET2*-mutated, but not *JAK2*-mutated, clones might be common initiating clones for ET and transformed AML. In a patient with *JAK2*-mutated ET, *SMARCC2*, *UBR4*, and *ZNF143*, but not *JAK2*, -mutated clones proliferated at AML transformation. Precise analysis using single-cell sorted CD34^+^/CD38^-^ fractions suggested that ET clone with *JAK2*-mutated and AML clone with *TP53* mutation was derived from the common clone with these mutations. Although further study is required to clarify the biological significance of *SMARCC2*, *UBR4*, and *ZNF143* mutations during disease progression of ET and AML transformation, the present results demonstrate the possibility of a common initial clone involved in both ET and transformed AML.

## Introduction

Essential thrombocythemia (ET) is a chronic myeloproliferative neoplasm (MPN) characterized by megakaryocyte hyperplasia, thrombocytosis, as well as thrombotic and hemorrhagic complications^1–3^. Although the median survival of ET patients is approximately 18 years, ET has the potential to transform into acute myeloid leukemia (AML)^4,5^. Since patients after transformation to AML have a dismal prognosis, it is necessary to clarify the biological mechanism for applying suitable therapeutic options.

Approximately 80% of ET patients have a mutation in one of the *JAK2*, *CALR*, or *MPL* gene, while these mutations are not associated with the risk of AML transformation^1,2,6^. Furthermore, up to 20% of ET patients are negative for all three mutations, referred to as triple-negative ET. It has been demonstrated that several additional genetic alterations, which are engaged in epigenetic regulation, cell-growth signaling, and RNA splicing machinery, cooperate with *JAK2*, *CALR*, or *MPL* mutation for the transformation to AML from ET^7–9^. The order of mutation acquisition also influences clonal evolution in MPNs, while it is not fully understood how clonal evolution occurs during disease progression in ET patients^7,9,10^.

On the other hand, both *JAK2* mutated- and unmutated-AML clones have been identified in transformed AML cells from *JAK2*-mutated MPNs, suggesting the presence of an initiating clone without *JAK2* mutation for ET and transformed AML^7,9,11^. Although several initiating mutations, such as *TET2* and *DNMT3A* mutations, have been identified in triple-negative ET patients, initiating clones without *JAK2*, *CALR*, and *MPL* mutations are not fully known. Moreover, the clonal evolution step from the initiating clone without *JAK2*, *CALR*, and *MPL* mutations is poorly understood.

The latent period of transformation to AML from ET is so long that it is difficult to compare the genetic status between transformed AML and ET at the initial diagnosis. In this study, we analyzed paired samples at the initial diagnosis and transformation to AML in eight ET patients, and identified three patients in whom *JAK2*-unmutated AML clones developed from *JAK2*-mutated ET in the chronic phase. We further investigated to identify initiating clones without *JAK2* mutation, and clarified the clonal evolution process during disease progression.

## Methods

### Patients and samples

Clinical characteristics of eight ET patients are shown in Table1 and Supplementary Table 3. The median duration from the diagnosis of ET until transformation to AML was 10.0 years (range: 1.5 – 20.0 years). Bone marrow (BM) or peripheral blood (PB) samples were obtained from each patient both at the ET phase and AML phase, and they were cryopreserved before use except for UPN2. At the initial diagnosis of UPN2, only DNA was available. From UPN8, BM cells at complete remission (CR) after chemotherapy for transformed AML and at relapse after achieving CR as well as a buccal swab were obtained. BM mononuclear cells at CR after chemotherapy for transformed AML were incubated with following antibodies before being sorted into hematopoietic stem cell (HSC) and hematopoietic progenitor cell (HPCs) fractions by a flow cytometer (FACSAria, BD Biosciences, San Jose, CA, USA): Human Lineage Cocktail 4 (CD2, CD3, CD4, CD7, CD8, CD10, CD11b, CD14, CD19, CD20, CD56, CD235a), anti-human CD34-APC (8G12), anti-human CD38-PE/Cy7 (HB7) (BD Biosciences), anti-human CD45RA-PerCP/Cy5.5 (HI100), anti-human CD123-PE (6H6) (BioLegend, San Diego, CA, USA) antibodies. HSCs were defined as lineage-marker (Lin)^-^CD34^+^CD38^-^fraction, HPCs were defined as Lin^-^CD34^+^CD38^+^CD123^+^CD45RA^-^ (common myeloid progenitor, CMP), Lin^-^CD34^+^CD38^+^CD123^+^CD45RA^+^ (granulocyte–macrophage progenitor, GMP), and Lin^-^CD34^+^CD38^+^CD123^-^CD45RA^-^ (megakaryocytic/erythroid progenitor, MEP) fractions.

**Table 1:** Patients’ characteristics.

UPN	Sex	Age (y. o.)	Treatment for ET	Duration to AML* (years)	Driver mutation in ET	Karyotype	
						ET	AML
**1**	M	70	HU	10.9	*CALR*^K385fs^^*^^47^	46,XY[20]	44,XY,-7,-16,-17, + mar[2]/ 44,idem,add(4)(q21)[18]
**2**	F	67	MCNU, BU	10.0	*CALR*^L367fs^^*^^43^	46,XX,der(15) t(1;15)(q23:q12-13)	44,XX,-5,del(8)(q22),add(17)(p11), + 18,psudic(18;9)(q23:p21) × 2[38]/ 46,XX,der(15)t(1;15)(q23:q12-13)[2]
**3**	M	92	HU	5.7	*MPL*^W515L^	ND	45,XY,del(1)(p?),der(16)t(16;17)(p13.1;q11.2),-17,add(21)(q22)[3]/ 45, idem,add(1p34),del(1),del(1)(p?)[6]
**4**	F	70	HU	15.6	*JAK2*^V617F^	46,XX[20]	44,XX,add(3)(p13),-5,add(6)(p21) ,-11,-13,-17, + 2mar[2] /45,idem, + mar[16]/46,XX[2]
**5**	F	75	HU	1.5	*JAK2*^V617F^	46,XX[20]	46,XX[20]
**6**	F	72	HU, MCNU	20.0	*JAK2*^V617F^	46,XX, + 1,der(1;13)(q10;q10)[5] /46,XY[5]	42,XX,-5,-7,i(11)(q10),-13,-14,-17 ,add(17)(q25),-18,-19, + 3mar[6]/ 41,idem,X,add(2)(q?23)[4]/ 46,XX, + 1,dr(1;13)(q10;q10)[1]
**7**	M	86	HU	6.1	*JAK2*^V617F^	ND	73 ~ 79,XXY, + Y, + 1,add(2)(p?13), + 8, + 9, + 15, + 16,-17, + 19, + 20, + 22, + 0 ~ 3mar,inc[cp9]/46,XY[1]
**8**	F	74	HU	9.9	*JAK2*^V617F^	46,XX[20]	43,XX,del(5)(q?),add(11)(p15), add(12)(p11.2),del(13)(q12q14), del(14)(q22q24),-17,-18, add(21)(q22),-22[1]/ 43, idem,-add(11), + add(11)(q23)[3]/ 43,idem,-add(11), + der(11)r(11;?)(p15q23;?)[9] /41 ~ 44,XX,del(5),-11,add(12) ,del(13),del(14),-17,-18,add(21),-22, + r, + 0 ~ 2mar[cp4]

*MCNU* ranimustine, *BU* busulfan, *HU* hydroxyurea, *ND* not determined.

*Duration from diagnosis of ET to transformation to AML.

In addition, we collected BM or peripheral blood mononuclear cells from 34 patients with ET in the chronic phase.

High-molecular-weight DNA and total RNA were extracted from each sample using QIAamp DNA Blood Mini Kit (QIAGEN, Hilden, Germany), QIAamp DNA Investigator Kit or QIAamp RNA Blood Mini Kit, and subjected to further analysis.

### Cytogenetic and molecular analyses

Cytogenetic G-banding analysis was performed using standard methods. Chimeric gene transcripts (Major *BCR::ABL1*, Minor *BCR::ABL1*, *PML::RARA*, *RUNX1::RUNX1T1*, *CBFB::MYH11*, *DEK::NUP214*, *NUP98::HOXA9*, *MLLT1::KMT2A*, *MLLT2::KMT2A, MLLT3::KMT2A,* and *MLLT4::KMT2A*) were examined at transformation of AML in all patients, as previously reported^12^.

Target sequencing of 54 genes, which are frequently identified in the presence of myeloid malignancies, was performed using TruSight Myeloid Sequencing Panel and Illumina MiSeq sequencer according to the manufacturer’s instructions (Illumina, San Diego, CA, USA) (Supplementary Table 1)^13–15^ in paired ET and AML samples of UPN1-8, CR and relapsed samples of UPN8. Sequence variation annotation was performed using known polymorphism databases, followed by mutation characterization, as previously reported^15^. Whole-exome sequencing (WES) was also performed on ET and transformed AML cells as well as a buccal swab in UPN8, as previously reported^16^. The variants detected in ET and/or AML samples but not in buccal swab were selected for further analysis. Each predicted variant sequence was confirmed by Sanger sequencing. The pathogenicity of variants were predicted with 5 pathogenicity tools: FATHMM^17^, LRT^18^, MutationTaster^19^, PolyPhen-2^20^ and SIFT^21^.

Copy-number abnormalities were identified using CNACS pipeline (https://github.com/OgawaLabTumPath/CNACS); the data of allele frequencies and sequenced depth of SNPs were used as input data^22^.

Mutations in whole coding regions of *ZNF143, UBR4*, and *SMARCC2* genes were analyzed in 40 patients with ET including UPN1-5 and UPN7. Whole exon regions of *ZNF143, UBR4,* and *SMARCC2* genes were amplified by the primer pairs indicated in Supplementary Table 2. Amplified products were subjected to mutation analysis using Nextera XT DNA Library Prep Kit and MiSeq sequencer according to the manufacturer’s instructions (Illumina).

### Single-cell mutation analysis

Lineage^-^/CD34^+^/CD38^-^ BM cells at CR after chemotherapy for transformed AML in UPN8 were sorted as single cells in a well of a 96-well plate. All processed 96 wells contained a single cell as verified by visual inspection under microscope.

Genomic DNA was extracted from each cell, and subsequently amplified by REPLI-g mini Kit (QIAGEN, Hilden, Germany). The DNA was analyzed for six mutations; *JAK2*^V617F^ in exon 14, *TP53*^R248W^, *TP53*^V173L^, *SMARCC2*^D381E^, *UBR4*^R450H^ and *ZNF143*^S286R^, and also a SNP within the *JAK2* c.2490G > A in exon 19, by direct Sanger sequencing of a PCR amplified region surrounding the target site with primer pairs indicated in Supplementary Table 2.

### Patient-derived xenograft model

Mononuclear cells isolated from fresh BM samples of UPN1 and UPN8 at transformation to AML were intravenously injected into 6-week-old NOD/Shi-scid, IL-2Rγnull (NOG) mice (purchased from the Central Institute for Experimental Animals, Tokyo, Japan) at 1 × 10^7^ viable cells per mouse, as previously reported^15^. T cells from patient BM samples were depleted by intraperitoneally injecting an anti-human CD3 (OKT3) antibody (Exbio Antibodies, Prague, Czech Republic). NOG mice were not pre-conditioned with irradiation. The engraftment of primary AML cells was monitored every 3 weeks in PB from the tail vein followed by flow cytometric analyses using FACSAria2 with anti-mouse CD45-PerCP (30-F11) (BioLegend), anti-human CD3-APC (UCHT1), and anti-human CD45-PE (HI30) antibodies (BD Biosciences). Mice were sacrificed when PB human CD45^+^ reached > 0.5% at 2 time-points, followed by flow cytometric assessment of BM for engrafted human cells with the same antibodies. The human CD45^+^ fraction was sorted from PDX BM by magnetic cell separation using MACS MicroBeads (human CD45 MicroBeads; Miltenyi Biotec, Bergisch Gladbach, Germany). Genomic DNA was extracted from unfractionated BM sample and subjected to targeted deep sequencing for 54 genes related to myeloid malignancies and *SMARCC2*, *UBR4*, and *ZNF143* as described in a previous section.

### Ethics approval

Informed consent for banking and further studies including genetic analysis of samples was obtained from all patients, and approval was obtained from the ethics committees of all participating institutions according to the Declaration of Helsinki. All methods were performed in accordance with relevant regulations and guidelines. All animal procedures were approved by the Institutional Animal Care and Use Committee of Nagoya University and carried out in accordance with the Regulations on Animal Experiments of Nagoya University and the ARRIVE guidelines.

## Results

### Genetic analysis in paired samples

Cytogenetic and RT-PCR analyses confirmed that no patients’ samples had t(9;22)(q34;q11)/*BCR::ABL1* abnormality. Mutation statuses of the patients at the initial diagnosis of ET and AML transformation are shown in Table 2. At ET, two patients (UPN1 and UPN2) showed *CALR* mutations; *MPL*^S505N^ mutations were also identified in one patient (UPN2), while both wild-type and mutant *MPL* mRNA were not expressed as previously reported^23^ Another patient (UPN3) had *MPL*^W515L^ and other five patients (UPN4-8) had *JAK2*^V617F^ mutation at ET. These driver mutations were detected at VAFs 18.4% to 75.1%. Additional mutations were identified at ET in seven out of eight patients: *TET2* or *TP53* mutations, three patients; *ASXL1* mutations, two patients; *EZH2* or *PHF6* mutation, one patient. These additional mutations were detected at a variety of VAFs (3.0—43.4%), most which were lower than VAFs of driver mutations. A part of ET clones with *CALR*, *JAK2* or *MPL* mutation or other clones without these driver mutations seemed to have additional mutations.

**Table 2:** Mutation status in ET and transformed AML cells.

UPN	Mutation	VAF (%)	
		ET	AML
**1**	*CALR*^K385fs^^*^^47^ *ASXL1*^G643fs^ *TP53*^C238S^ *U2AF1*^Q157R^	22.8 5.1 - -	34.2 4.7 37.4 33.2
**2**	*CALR*^L367fs^^*^^43^ *MPL*^S505N^ *TP53*^C153Y^ *NRAS*^Q61K^	51.0 39.0 3.0 -	47.0 45.0 93.0 2.0
**3**	*MPL*^W515L^ *TP53*^R248Q^ *EZH2*^G660R^ *PHF6*^G306E^	75.1 14.1 18.1 27.8	88.0 83.9 40.1 -
**4**	*JAK2*^V617F^ *TP53*^H178P^	18.4 -	95.7 96.7
**5**	*JAK2*^V617F^ *TET2*^Q1030^^*^ *TET2*^C1271fs^ *ASXL1*^E773^^*^ *RUNX1*^L98fs^ *CEBPA*^P23fs^	32.5 40.3 36.0 37.3 - -	50.1 49.6 47.6 53.1 46.6 40.8
**6**	*JAK2*^V617F^ *TET2*^Q278^^*^ *TP53*^M133K^ *IDH2*^R140Q^ *NRAS*^G12S^	68.5 10.3 - - -	- 48.3 82.4 45.5 6.4
**7**	*JAK2*^V617F^ *TET2*^R1262L^ *TET2*^H1380Y^ *TP53*^R280S^ *SRSF2*^P95R^ *CBL*^G413D^	84.0 41.8 43.4 - - -	9.9 60.0 34.2 77.6 73.8 67.1
**8**	*JAK2*^V617F^ *TP53*^R248W^ *TP53*^V173L^ *SMARCC2*^D381E^ *UBR4*^R450H^ *ZNF143*^S286R^	40.5 4.7 4.4 50.7 47.7 47.9	2.7 87.5 3.5 47.9 48.5 60.6

- indicates not detected

**Figure 1:**
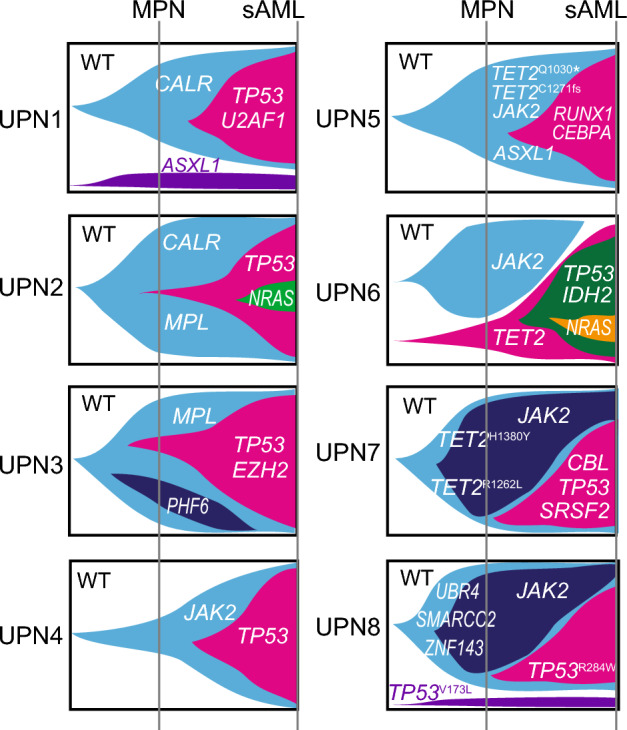
Serial mutational spectrum from ET to transformed AML. The serial mutational spectrum of each patient in the clinical course from ET to transformed AML is shown in fish plot format. Although five patients (UPN1-5) had the same *CALR*, *MPL*, or *JAK2* mutation as that of ET at AML transformation, three patients (UPN6-8) in whom the *JAK2*^V617F^-mutated clone was dominant at ET showed that the dominant clones at AML transformation did not have *JAK2*^V617F^ mutation.

At AML transformation, five patients (UPN1-5) had the same *CALR*, *MPL*, or *JAK2* mutation as that of ET along with new additional mutations: *TP53*, *NRAS*, *U2AF1*, *RUNX1*, or *CEBPA* mutation. These results indicated that ET and transformed AML cells developed from the same *CALR*, *MPL*, or *JAK2*-mutated initiating clone acquiring additional mutations in UPN1-5 (Fig. 1).

Notably, three patients (UPN6-8) in whom the *JAK2*^V617F^-mutated clone was dominant at ET showed that the dominant clones at AML transformation did not have the *JAK2*^V617F^ mutation. In two patients with *JAK2*^V617F^ and *TET2* mutation(s) at ET (UPN6 and UPN7), *JAK2*^V617F^ mutation was not detected or detected at low VAF (9.9%) in transformed AML cells; instead VAFs of *TET2* mutation were detected at almost same or increased level compared with ET. Furthermore, *TP53, IDH2, NRAS, SRSF2* and *CBL* mutations were additionally identified in transformed AML cells. These results indicated that *TET2*-mutated, but not *JAK2*-mutated, clones were the common initiating clones in UPN6 and UPN7 (Fig. 1). However, in transformed AML of UPN8, VAF of *TP53*^R248W^ mutation increased to 87.5 from 4.7%, while *JAK2*^V617F^ mutation decreased to 2.7 from 40.5% and *TP53*^V173L^ mutation was stable at VAF below 5%; no other mutations common to both ET and AML were detected. UPN8 at AML transformation had acquired a complex karyotype including numerical or structural abnormalities of chromosome 5 and 7, which is supposed to be involved in the transformation (Table 1).

*TP53* mutations were detected seven of eight patients at AML with a VAF 37.4% in one patient whose blast rate was 10% in the sample and over 80% in other patients. The deletion of chromosome 17 resulting in the loss of heterozygosity (LOH) at *TP53* locus were detected in six of these patients (Table 1). *TP53* mutations were already detected in ET samples in three patients using target sequencing with 1.0% as cutoff VAFs for mutations; whereas the possibility that the minor clones with *TP53* mutations at VAF lower than 1.0% had existed in other four patients at ET has not been ruled out.

We showed a model of clonal changes from ET to AML in each patient assumed by VAFs of analyzed genes (Fig. 1); other models can be also drafted. Therefore, a single cell analysis was performed to identify detailed clonal dynamics in UPN8 as described in a later section.

### Search for initiating mutations by Whole-exome sequence

The UPN8 patient received chemotherapy after AML transformation, and achieved complete remission (CR); however, the patient subsequently showed relapsed AML. We also analyzed the mutation status at CR and relapse using BM samples. Mutation statuses of *JAK2*^V617F^, *TP53*^R248W^, and *TP53*^V173L^ in BM samples at CR and relapse were almost the same as those at the initial diagnosis of ET and AML transformation, respectively (Table 3). These results suggest that ET clones with *JAK2*^V617F^ were dominant at CR and AML clones with *TP53*^R248W^ re-increased at relapse, and moreover that the initiating clone with genetic mutations other than these mutations could exist. Therefore, we performed WES analysis to search for initiating mutations in samples at ET or AML phase in UPN8.

**Table 3:** Mutation status during the disease course in UPN8.

Mutation	VAF (%)				karyotype	
	MPN	sAML	CR	Rel	CR	Rel
*JAK2 *^V617F^	40.5	2.7	39.8	7.5	46,XX[20]	complex*
*TP53*^R248W^	4.7	87.5	8.4	63.1		
*TP53*^V173L^	4.4	3.5	8.4	7.1		
*SMARCC2*^*D381E*^	50.7	47.9	47.0	43.7		
*UBR4*^R450H^	47.7	48.5	43.4	43.2		
*ZNF143*^S286R^	47.9	60.6	43.2	29.6		

*VAF* variant allele frequency, *CR* complete response, *Rel* relapse.

*43, XX, del(5)(q?), add(11)r(11;?)(p15q23;?), add(12)(p11.2), del(13)(q13q14), del(14)(q22q24), -17, -18, add(21)(q22), -22[11] /43, idem, + add(11)(q23), -der(11)r(11;?), -15, + mar[1] /40 ~ 43, XX, del(5), der(11)add(11)(p15)add(11)(q23), add(12), del(13), del(14), -17, -18, add(21), -22[cp6] /46, XX[1].

**Figure 2:**
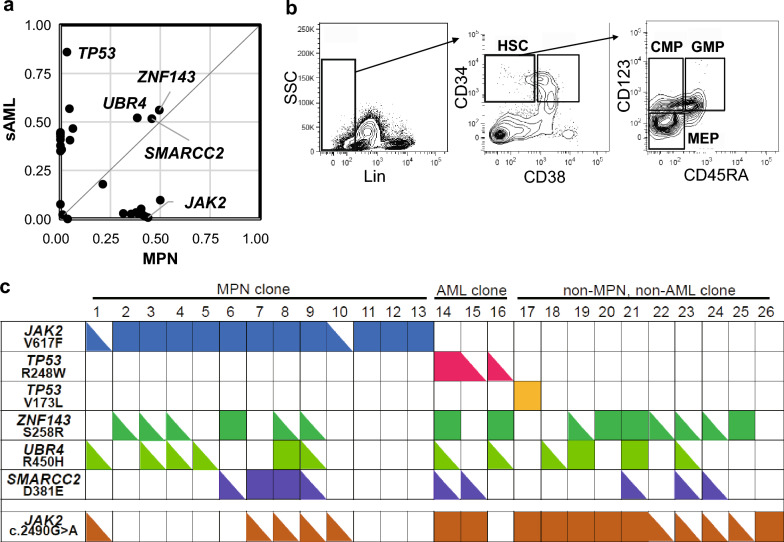
Clonal evolution in UPN8. (**A**) Comparison of VAFs of mutated genes detected by whole-exome sequence analysis between ET and transformed AML cells. *ZNF143*^S286R^, *UBR4*^*R*450H^, and *SMARCC2*^D381E^ mutations were identified at the same VAF levels in ET and transformed AML cells. (**B**) Mutation analysis in HSC and HPCs. Mutation statuses of *JAK2*^V617F^, *TP53*^R248W^, *ZNF143*^S286R^, *UBR4*^*R*450H^, and *SMARCC2*^D381E^ in HSC, CMP, GMP, and MEP fractions were analyzed. (**C**) Single-cell mutation analysis of BM samples at CR after chemotherapy for transformed AML. CD34^+^/CD38^-^ cells were sorted as single cells, and we analyzed *JAK2*^V617F^, *TP53*^R248W^, *TP53*^V173L^, *ZNF143*^S286R^, *UBR4*^R450H^, and *SMARCC2*^D381E^ mutations. The closed square and triangle indicate homozygous and heterozygous mutations, respectively.

WES analysis revealed that *ZNF143*^S286R^, *UBR4*^*R*450H^, and *SMARCC2*^D381E^ mutations were commonly identified in both ET and transformed AML cells (Fig. 2a). These mutations were not identified in the buccal swab sample indicating that these were somatic mutations (Supplementary Fig. 1a). These mutations were classified as pathogenic in most of the in-silico prediction tools (Supplementary Table 4). We also interrogate The Cancer Genome Atlas (TCGA) database, which comprises 10,967 samples across 32 different cancer types^24^ for exploring the possibility that these mutations are single nucleotide polymorphisms. *UBR4*^*R*450H^ mutation had been detected in one patient with endothelial cancer as single nucleotide variation with unknown significance and neither of *ZNF143*^S286R^ or *SMARCC2*^D381E^ mutation had been detected. WES showed VAFs of *ZNF143*^S286R^, *UBR4*^*R*450H^, and *SMARCC2*^D381E^ mutations at ET and AML were 47.9 and 60.6%, 47.7 and 48.5%, and 50.7 and 47.9%, respectively. Furthermore, VAFs of *ZNF143*^S286R^, *UBR4*^*R*450H^, and *SMARCC2*^D381E^ mutations at CR and relapse were similar to those at ET or AML phase (Table 3). These results suggest that cells harboring these three somatic mutations can be common ancestors of both ET and AML.

### Mutation analysis in HSC and HPC fractions

We then analyzed the mutation status in HSC and HPCs (CMP, GMP, and MEP) fractions to clarify the pattern of mutational acquisition throughout the ET- and AML-phase in UPN8. We used the BM sample at CR after chemotherapy for transformed AML, which was thought to be composed of ET-, AML- and normal cells. *JAK2*^V617F^, *ZNF143*^S286R^, *UBR4*^*R*450H^, and *SMARCC2*^D381E^ mutations were identified in HSC and HPCs fractions (Fig. 2b) at similar VAFs to whole mononuclear cells (MNC) (Table 4), suggesting that ET clones had already acquired these mutations at hematopoietic stem cell stage and differentiated to progenitor cells. In contrast, *TP53*^R248W^ mutation was identified in the HSC fraction at a higher VAF compared to MNC and HPCs fraction. Given that *TP53*^R248W^ mutation was detected in the AML sample at a much higher VAF compared to ET sample, these results suggest that AML clones, which were concentrated into the HSC fraction, did have acquired *TP53*^R248W^. The failure to detect *TP53*^R248W^ mutation in the CMP fraction may be attributed to an inability of the current methods rather than to an actual absence of this mutation considering that the mutation was detected in both GMP and MEP fractions.

**Table 4:** Mutational status in HSC, HPC fractions at complete remission in UPN8.

Mutation	VAF (%)				
	MNC	HSC	CMP	GMP	MEP
*JAK2 *^V617F^	42.2	39.0	51.1	48.8	41.0
*TP53*^R248W^	8.8	18.5	0	13.0	12.9
*TP53*^V173L^	13.0	13.6	21.4	17.4	7.1
*SMARCC2*^*D381E*^	42.3	34.9	57.5	45.9	52.1
*UBR4*^R450H^	43.4	44.2	69.7	56.1	42.4
*ZNF143*^S286R^	48.7	40.0	78.1	63.3	57.1

*MNC* mononuclear cells, *HSC* hematopoietic stem cell, *HPCs* hematopoietic progenitor cell, *CMP* common myeloid progenitor, *GMP* granulocyte–macrophage progenitor, *MEP* megakaryocytic/erythroid progenitor.

### Single-cell mutation analysis

We further explored the common initiating clone for ET and AML in UPN8 using single-cell mutation analysis. The CD34^+^/CD38^-^ cells in BM samples at CR after chemotherapy for transformed AML were sorted as single cells and analyzed for *JAK2*^V617F^, *TP53*^R248W^, *TP53*^V173L^, *ZNF143*^S286R^, *UBR4*^R450H^, and *SMARCC2*^D381E^ mutations. We analyzed 96 single-cells and obtained results in 26 single-cells (Fig. 2c). Contrary to expectations from the VAF in the target sequencing and WES, *JAK2*^V617F^ mutation was identified only in 13 of the 26 (50.0%) cells, and 11 of them were homozygous. We investigated whether the cells with *JAK2*^V617F^ in exon 14 had LOH on *JAK2* locus even though copy number change or imbalance was not observed on *JAK2* locus (Supplementary Fig. 1b). Analysis of the SNP within the *JAK2* exon 19, c.2490G > A, revealed that most of cells with homozygous *JAK2*^V617F^ had only c.2490G allele at the SNP site (2–6 and 11–13 in Fig. 1c) , most of cells with wild type *JAK2* had c.2490A allele (14–15, 17–21 and 25 in Fig. 1c), and all two cells with heterozygous *JAK2*^V617F^ (1 and 10 in Fig. 1c) and some cells with homozygous *JAK2*^V617F^ or *JAK2*^WT^ had heterozygous SNP (7–9 or 22–25 in Fig. 1c). These data support the view that homozygous *JAK2*^V617F^ was caused by mitotic recombination leading to acquired uniparental disomy (UPD) on chromosome 9p without copy number change on *JAK2* locus, which had been reported in MPNs^25^. The patterns of the *JAK2* SNP in some cells, e.g. clone 16 in Fig. 1c, were not compatible with the model. None of the cells with *TP53*^R248W^ mutation harbored *JAK2*^V617F^ mutation, which suggests AML clones with *TP53*^R248W^ mutation are derived from cells without *JAK2*^V617F^ mutation as expected from the VAFs in the bulk sequencing analyses. *ZNF143*^S286R^, *UBR4*^R450H^ and *SMARCC2*^D381E^ mutations were identified both in *JAK2*^V617F^-mutated and -nonmutated cells and also in cells with *TP53*^R248W^ mutation but not in the cell with *TP53*^V173L^. These results suggest that ET clone with *JAK2*^V617F^ and AML clone with *TP53*^R248W^ could be derived from the common initial clone with *ZNF143*^S286R^, *UBR4*^R450H^, and *SMARCC2*^D381E^ mutations, and clones with *TP53*^V173L^ could be derived from another clone without these three mutations. However, the mutational pattern of *ZNF143*^S286R^, *UBR4*^R450H^, and *SMARCC2*^D381E^ was diverse both in *JAK2*^V617F^-mutated and – unmutated cells; only a part of the *JAK2*^V617F^-mutated cells (nine of the 13) and un-mutated cells (11 of 13) were co-mutated with *ZNF143*^S286R^ *UBR4*^R450H^, or *SMARCC2*^D381E^ mutation, and the numbers of co-mutations also varied between cells (Fig. 2c).

### Engrafted clone in patient-derived xenograft model

Our previous study had shown that AML-PDX models are useful for analyzing the clonal dynamics and that chemotherapy-resistant clones dominantly engraft in AML-PDX models even when they are minor in primary AML^15^. AML transformed from MPNs are often refractory to chemotherapy, and serial mutational analysis in this study has shown that some transformed AML samples contain multiple clones. We then tried to identify the clones which effectively graft and propagate in the PDX model. We inoculated NOG mice with transformed AML cells of three patients (UPN1, -2 and -8) and established two AML-PDX models from UPN1 and -8. (Table 5). In UPN1, engrafted cells harbored *CALR*^K385fs47^, *TP53*^C238S^ and *U2AF1*^Q157R^ mutations, but not *ASXL1*^G643fs^ mutation. Furthermore, VAFs of *CALR*^K385fs47^ (40.2%) and *U2AF1*^Q157R^ (49.5%) mutations in the engrafted cells were the same as primary AML cells, while that of *TP53* mutation increased to 99.9 from 37.4% in primary AML cells. These results suggest that a major clone in AML with *CALR*^K385fs47^, *TP53*^C238S^ and *U2AF1*^Q157R^ with *TP53* LOH which is attributed to deletion of chromosome 17 engrafted and propagated in PDX. In UPN8, the engrafted cells harbored *TP53*^R248W^ (VAF, 98.7%), *ZNF143*^S286R^ (VAF, 52.2%), *UBR4*^R450H^ (VAF, 50.4%), and *SMARCC2*^D381E^ (VAF, 44.8%) mutations at the same VAFs as the primary AML cells, but not *JAK2*^V617F^ or *TP53*^V173L^ mutations. These results suggest that a major clone in AML with *TP53*^R248W^, *ZNF143*^S286R^, *UBR4*^R450H^, and *SMARCC2*^D381E^ engrafted in PDX.

**Table 5:** Comparison of mutation status between AML and PDX cells.

UPN	Mutation	VAF (%)	
		AML	PDX
1	*CALR*^K385fs*47^	34.2	40.2
	*ASXL1*^G643fs^	4.7	-
	*TP53*^C238S^	37.4	99.9
	*U2AF1*^Q157R^	33.2	49.5
8	*JAK2*^V617F^	2.7	-
	*TP53*^R248W^	87.5	98.7
	*TP53*^V173L^	3.5	-
	*SMARCC2*^D381E^	47.9	44.8
	*UBR4*^R450H^	48.5	50.4
	*ZNF143*^S286R^	60.6	52.2

- indicates not detected

### Screening of *ZNF143*, *UBR4*, and* SMARCC2* mutations in ET or other myeloid neoplasms

Lastly, we screened whole coding regions of *ZNF143, UBR4*, and *SMARCC2* genes in 40 patients with ET including UPN1-5 and UPN7. However, we did not find mutations in any of the patients. An additional interrogation of Beat AML database(n = 672)^26^ and data from other 10 studies targeting myeloid neoplasms (n = 9,889)^27–32^ using cBioPortal^33,34^ revealed that *ZNF143*^S286R^ mutation had been detected in one patient with ET as a somatic mutation^32^. Neither *UBR4*^R450H^ nor *SMARCC2*^D381E^ had been detected in the database.

## Discussion

We found that five of the eight patients showed the proliferation of *JAK2*, *CALR*, or *MPL*-mutated clones with additional mutations at AML transformation. Particularly, *TP53* co-mutated clones dominantly proliferated in four of the five patients. Although the transformed AML cells did not harbor *TP53* mutation in one patient (UPN5), they had additional mutations in *TET2*, *ASXL1*, *RUNX1*, and *CEBPA* genes. These results indicated that the additional mutations, particularly *TP53* mutation, drove the *JAK2*, *CALR*, or *MPL*-mutated clone in the chronic phase of ET to AML transformation.

In contrast, the *JAK2*, *CALR*, or *MPL*-unmutated clone proliferated at AML transformation in three patients (UPN6-8). Notably, proliferated clones in transformed AML already existed at the initial diagnosis of ET in all patients. In UPN6 and 7, *TET2*-mutated clones were identified both at the initial diagnosis and AML transformation, while *TP53*, *IDH2*, and *NRAS* mutations in UPN6 and *TP53*, *SRSF2*, and *CBL* mutations in UPN7, which were additionally identified in transformed AML, were not detected at the initial diagnosis. These results indicated that *TET2*-mutated, but not *JAK2*-mutated, clones were common initiating clones for ET and transformed AML. Interestingly, VAF of *NRAS*^G12S^ mutation (6.4%) was much lower than the other mutations at AML transformation in UPN6. The *NRAS* co-mutated minor clone was also observed in transformed AML cells of UPN2. We reported that the Marimo cell line, which harbors *CALR*^L367fs*43^, *MPL*^S505N^, *TP53*^C153Y^, and *NRAS*^Q61K^ mutations, was established from the *NRAS* co-mutated clone in UPN2^23,35^. These results support the suggestion that *NRAS* mutation provided further growth advantage to the transformed AML clone even with *TP53* mutation.

In UPN8, *SMARCC2*, *UBR4*, and *ZNF143* mutations as well as *JAK2* and *TP53* mutations were identified at the ET phase, while *SMARCC2*, *UBR4*, *ZNF143,* and *TP53*, but not *JAK2*, -mutated clones proliferated at transformation to AML. Since VAF of *TP53* mutation increased to 87.5% in transformed AML cells, transition from heterozygosity to homozygosity in *TP53* mutation might be associated with evolution to AML, as previously reported^7,8,36^ However, the effect of the pathophysiology of *SMARCC2*, *UBR4*, and *ZNF143* mutations on the development and progression of ET is, to date, unclear. Each VAF of identified gene mutation was almost the same among HSC and HPC fractions in the CR state after chemotherapy for transformed AML. Furthermore, VAF of *JAK2* mutation was the same as that of *SMARCC2*, *UBR4*, or *ZNF143* mutation, and that of *TP53* mutation was lower than the other mutations. Mutation analysis in the single cells sorted from the CD34^+^/CD38^-^ fraction revealed that both *JAK2*^V617F^ and *JAK2* wild-type cells including *TP53*^R248W^-mutated cells had *ZNF143*, *UBR4*, and *SMARCC2* mutation. These results suggest that ET clone and AML clone could be derived from the common initial clone harboring *ZNF143*, *UBR4*, and *SMARCC2* mutations, although their biological significance is unclear. The further analysis of the SNP in *JAK2* leaded us to surmise that most of cells in the fraction at CR had UPD on chromosome 9p resulting in homozygous *JAK2* mutation with LOH. One model for the clonal change in UPN8 can be as follows. A part of clone with *ZNF143*, *UBR4*, and *SMARCC2* gained *JAK2*^V617F^, underwent mitotic recombination on chromosome 9p and propagated as ET clones; and another clone harboring the three mutations gained *TP53*^R248W^ in ET phase and evolved into AML clones. On the other hand, this model is not applicable to all cells. Some cells *JAK2*^V617F^ had none of these three mutations and mutational pattern including zygosity of each variant varied between cells. This is a limitation of the single-cell mutation analysis by Sanger sequencing in this study, which warrant consideration.

PDX-model analysis of AML cells from UPN1 and UPN8 clarified that the transformation-associated clone had a growth advantage. In a NOG mouse inoculated with AML cells of UPN8, engrafted AML cells consisted of *ZNF143, SMARCC2, UBR4,* and *TP53*-mutated clones, but not *JAK2*-mutated clones. *ZNF143, SMARCC2,* and *UBR4* mutations were not identified in 40 ET patients in this study; however, *ZNF143*^S286R^ mutation has been reported in ET patients^32,37^. It is possible that these mutations are cooperatively involved in the mechanisms of disease initiation and evolution in this patient based on their known biological functions. Further study is required to clarify the biological mechanism of these mutations in the pathophysiology of ET.

### Supplementary Information

Supplementary Information.

## Data Availability

The authors will supply the relevant data in response to reasonable requests.
