# Supplementary material for: Initiating-clone analysis in patients with acute myeloid leukemia secondary to essential thrombocythemia
Source: Sci Rep. 2024 Jul 10;14:15906. doi: 10.1038/s41598-024-66461-8 (PMC11237009; doi:10.1038/s41598-024-66461-8)

Supplementary Information

Initiating-clone analysis in patients with acute myeloid leukemia secondary to essential thrombocythemia

Yoko Ushijima,[1*] Seara Naruse,[1*] Yuichi Ishikawa,[1] Naomi Kawashima,[1] Masashi Sanada,[2] Marie Nakashima[1], Jeong Hui Kim[1], Seitaro Terakura[1], Rika Kihara[3,4], Koichi Watamoto[4], Takahiro Nishiyama[5], Kunio Kitamura[5], Tadashi Matsushita[6], and Hitoshi Kiyoi[1]

[1] Department of Hematology and Oncology, Nagoya University Graduate School of Medicine, Nagoya, Japan

[2] Clinical Research Center, National Hospital Organization Nagoya Medical Center, Nagoya, Japan

[3] Department of Hematology and Oncology, Konan Kosei Hospital, Konan, Japan

[4] Department of Hematology, Komaki City Hospital, Komaki, Japan

[5] Division of Hematology, Ichinomiya Municipal Hospital, Ichinomiya, Japan

[6] Department of Transfusion Medicine, Nagoya University Hospital, Nagoya, Japan

Statement of equal author contribution: *Yoko Ushijima and Seara Naruse contributed equally to this study as first authors.

Supplementary Table 1. Analyzed gene list

ABL1	CEBPA	HRAS	MYD88	SF3B1
ASXL1	CSF3R	IDH1	NOTCH1	SMC1A
ATRX	CUX1	IDH2	NPM1	SMC3
BCOR	DNMT3A	IKZF1	NRAS	SRSF2
BCORL1	ETV6/TEL	JAK2	PDGFRA	STAG2
BRAF	EZH2	JAK3	PHF6	TET2
CALR	FBXW7	KDM6A	PTEN	TP53
CBL	FLT3	KIT	PTPN11	U2AF1
CBLB	GATA1	KRAS	RAD21	WT1
CBLC	GATA2	MLL	RUNX1	ZRSR2
CDKN2A	GNAS	MPL	SETBP1	

Supplementary Table 2. Primer sequences for target sequencing or Sanger sequencing

target sequencing		
SMARCC2	Forward	5’-TCTGAAAAGTTTAAATGGGGAGCTG-3’
	Reverse	5’-GAGACAAAGACGACACCAGGAAGAG-3
	Forward	5’-GTGGAAACATAGAAGTAGGGCCAGG-3’
	Reverse	5’-CAGGCCTACATGCTACTTTTTGCAA-3
	Forward	5’-TTGAGTCTGCATTGTGCCATGATTG-3’
	Reverse	5’-AGCGGCTTAACAGTACAAGTACATA-3
	Forward	5’-TTAGGGCTGCCACCAAGAAG-3’
	Reverse	5’-TCTTTCCCAGTGAGCCCTCT-3
	Forward	5’-ATTGCCAAGGTCCTCATGGTAGAAC-3’
	Reverse	5’-GGCAAGCCACATAACCTTCTTCCTA-3
	Forward	5’-TGTCCAGCTCCTAAGAGAACCTATAAAGA-3’
	Reverse	5’-CAGTGAGCCGAGATCTGACAGAGC-3
	Forward	5’-CCAGCCTCCATGCTTGTTTATTTAT-3’
	Reverse	5’-ATGTCCTCCAAAGACAAAATGGTTC-3
	Forward	5’-GCACATCAAGTTTCTAAGTCCTGAG-3’
	Reverse	5’-TCAGCTGAAGTTCTCATAGTCTTGG-3
UBR4	Forward	5’-AGTGCTAAGGGAATACGGATCAGTG-3’
	Reverse	5’-AGGAGTGCAACGTAAAAAGAGGTCA-3
	Forward	5’-TTGAGTATTCTGGTCTGCCCCTTTT-3’
	Reverse	5’-CAGCTAGGACTGAGGAGGCTAAGG-3
	Forward	5’-AGATTGCACACTCCTGGTTTAGTGG-3’
	Reverse	5’-GCTTCAAAGGCAGTAAGATCAACAGG-3
	Forward	5’-CTGGTAACACCACTGATCTCCACTG-3’
	Reverse	5’-TATTACAGGAAGCAAACCAGGAGGC-3
	Forward	5’-AGCTGGTCTAAAATTGCTGGGCATA-3’
	Reverse	5’-AGGAGTTTCTGTTTGTTTTTGTGGGT-3
	Forward	5’-AGAATTTCTCTGCGAATACTGCGGA-3’

	Reverse	5’-TAGACGGGATCTACTCAGGCTGATC-3
	Forward	5’-AGCTAAATTTCCTTTTGGGTCTTGG-3’
	Reverse	5’-CATAGAACAGGAGTGAACACTGAGA-3
	Forward	5’-AAGATAGAGGGCTGTTGTTTGAGGC-3’
	Reverse	5’-GAGTTCAAGACCAGCCTACACAACA-3
	Forward	5’-GACCTTGGTAGTTTAGGCCACATGG-3’
	Reverse	5’-TTCCTAGCCACCTATGCCAGTTCAT-3
	Forward	5’-AGGCAAGCATGAACTATCTGCAATG-3’
	Reverse	5’-GTAGCTGGGACTATAGTTGCTGTGC-3
	Forward	5’-GGGGCCCATAAAGTTGCTGATTTTT-3’
	Reverse	5’-GCTAACAGAGTAAGTCTCCCCAGGA-3
	Forward	5’-AGGAGGAAGAAGAGGCTTATGGTGT-3’
	Reverse	5’-ATGAGCTCAGAATGGAAGGGAGGAT-3
	Forward	5’- AGAGGATGGTTTTTACCTTTCCCTT-3’
	Reverse	5’-AGATACTTGTGCCTTTTGAGCTGGC-3
	Forward	5’-TCATCACCTGAGGTTGGGGGATATT-3’
	Reverse	5’-AACCCTCTCTCTCAAGCTCACAGAT-3
	Forward	5’- TTCAGATCTCTCAGCAGGTGTCAGT-3’
	Reverse	5’-ACTGCTGACCTCAACAAATTCAACA-3
	Forward	5’- TACAGAAGGTCAGCTGTATATCTGC-3’
	Reverse	5’-ATCGTATTCCTACCCTACTCAAAGC-3
	Forward	5’- ACAGATTCTTTTTATGGGACCACAC-3’
	Reverse	5’-ACCAATGTACTCAGTGTCATTTCAC-3
	Forward	5’- GTTCACATTCCCCCAGTTAATGATC-3’
	Reverse	5’-GAGCAAGAGCATAGCTGTCTTAATC-3
	Forward	5’- GTTTATCTTTCCAAGGTTGCTGTCA-3’
	Reverse	5’-ATACTGAATAAGCCCCAAAGTCCTT-3
	Forward	5’- GAAAGAGTTGTGACCAAGACTGAGA-3’
	Reverse	5’-TACTTTAGCTAGAGAGAGGAGGGAG-3
	Forward	5’- TGAAAGTATTCAGCATATAAGTGTCC-3’
	Reverse	5’-GGTAGATTAAACATTTGCACACAGTG-3

	Forward	5’-CTCAAAGTGCTCACAAGACCTTATT-3’
	Reverse	5’-GGCCCATAAAAGCTGAGAACTAATC-3
	Forward	5’- TGATTGGAGACAGTTTCTACTCCTG-3’
	Reverse	5’-TGATTCCACCAGTAACCAACTATGT-3
	Forward	5’-GCTATGCCTGTAATTTCCTCCATTC-3’
	Reverse	5’-AAAGGACCCACATGATTTCTCTGTA-3
	Forward	5’-CCTCTAGTATCCCTAGCCTGAGAAA-3’
	Reverse	5’-AGAAAGTTCTGATTTTCTCCCCAGT-3
	Forward	5’-AGCAAGGGAATATCAAGAAGTCACT-3’
	Reverse	5’-TCTGGAAAGTAGAAGTAGCCGCTG-3
	Forward	5’-TCACTCACTAGCTTGCTGACTTTGG-3’
	Reverse	5’-CTCACTCAGGGCAACAGAGCTTATC-3
	Forward	5’-TTTGAGTTGGTTCCTTTCATCATCG-3’
	Reverse	5’-TGTCAATAGCAGCCACTCTCTTTAT-3
ZNF143	Forward	5’-GAATTTCAGCAGGAGAGGAAGGCTC-3’
	Reverse	5’-AAGAAAGGGTGAGGGGGAAACAATC-3
	Forward	5’-GTAATGCAGGTGAGAGACTGGTTGT-3’
	Reverse	5’-AAAAACTAGCTGGGAGAGGCAGCAT-3
	Forward	5’-AGTCACAGGTTCATTGTAACTGGCT-3’
	Reverse	5’-AGTGGTCATGTTAATGCTCAAGGTT-3
	Forward	5’-GATGACCTGAGATTGGAAACCCACA-3’
	Reverse	5’-GGGCCACTGTTAATCTTTTCTGGGA-3
	Forward	5’-GGCGAGTATTTCCAAGCTCCCATAA-3’
	Reverse	5’-AAATGCTTCTCCCATTCCTGCAGAA-3
	Forward	5’-AACTGGGCAACATCTCCATCCTTTT-3’
	Reverse	5’-GTGCCATCATATGCTGATCACCTCA-3
	Forward	5’-AGGGAAGGGTTAGACTGGATACCTG-3’
	Reverse	5’-CCAGCCTGTCTCAAAAACCAGAAGA-3
	Forward	5’-GCTGTTTGTCAGTAGTACCACTGGT-3’
	Reverse	5’-GAAAGTCTGGGTTCTCGTCTTGGTC-3
**Sanger sequencing**		

JAK2 V617F	Forward	5’-CAAAGTTCAATGAGTTGACCCCTA-3’
	Reverse	5’-ACACAAGGTTGGCATATTTTTCATAAG-3’
TP53	Forward	5’-TGTTCACTTGTGCCCTGACT-3’
	Reverse	5’-CAGCCCTGTCGTCTCTCCAG-3’
	Forward	5’-CTGCTTGCCACAGGTCTCC-3’
	Reverse	5’-GGATGTGATGAGAGGTGGATGG-3’
SMARCC2	Forward	5’-GGCTTTTGGTAGGGCTTTCT-3’
	Reverse	5’-ATGCAGGTCTGGATTCTTGG-3’
UBR4	Forward	5’-TTGTGCCTTTCACAACAGCA-3’
	Reverse	5’-AGCCAGGTAGGAAGGCAAAT-3
ZNF143	Forward	5’-CTCAGTGTGCATGTTTAGAGCA-3’
	Reverse	5’-ACACTCTGTTTCCACTAGTACCT-3
JAK2 c.2490G>A	Forward	5’-AGTTTAGTCCAGAGAATGTTATTTGCT-3’
	Reverse	5’-ACCTTGCCAAGTTGCTGTAGA-3’

Supplementary Table 3.

Additional information about Patients' characteristics.

UPN	ET				AML	
	WBC [/μL]	Hb [g/dL]	Hct [%]	Plt [10^3/μL]	Origin of analyzed sample	Blast[%]
**1**	5,500	14.3	42.5	1,317	PB	10
**2**	NA	NA	NA	1,200	BM	82
**3**	7,500	12.7	38.1	839	BM	51
**4**	15,600	15.2	45.0	948	BM	28
**5**	12,600	13.3	41.1	665	BM	41.2
**6**	7,100	13.3	40.5	626	BM	69.5
**7**	17,900	13.1	41.6	800	PB	32
**8**	12,400	12.8	42.1	1,133	BM	21.5

NA, Not Available;

Supplementary Table 4.

Pathogenicity scores for the *SMARCC2*, *ZNF143*, *UBR4* mutations

Mutation	FATHMM		LRT		Mutation Taster		PolyPhen-2		SIFT	
	score	pred.	score	pred.	score	pred.	score	pred.	score	pred.
SMARCC2^{D381E}	0.63	T	0	D	1	D	0.971	D	0.12	T
UBR4^{R450H}	1.82	T	0	D	1	D	0.964	D	0.02	D
ZNF143^{S286R}	2.74	T	0	D	1	D	0.609	P	0.23	T

pred.: prediction, D: deleterious/ probably damaging/ disease causing, P: possibly damaging, T: tolerated

Supplementary figure 1

(a) Integrative Genomics Viewer (IGV) view of *SMARCC2*^{D381}, *UBR4*^{R450} and *ZNF143*^{S286R} in ET sample, AML sample or germline (buccal swab) sample.

(b) Total copy number (CN) and allele-specific copy number (AsCN) in ET or AML sample.

Supplementary Fig. 1

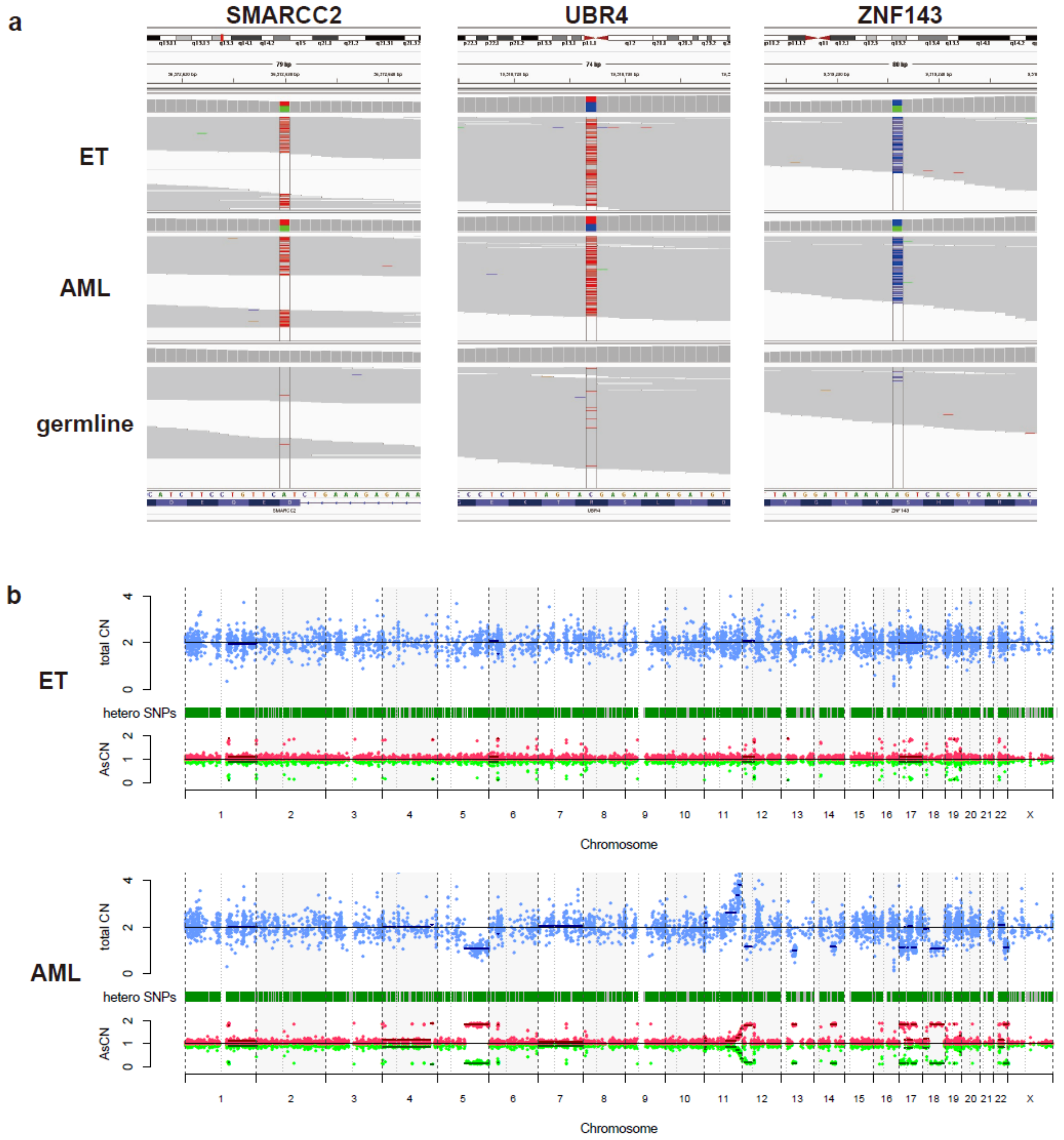

Supplement: Supplementary file 1 — Supplementary Information. [file 41598_2024_66461_MOESM1_ESM.pdf]
